# Simultaneous Assessment of Acidogenesis-Mitigation and Specific Bacterial Growth-Inhibition by Dentifrices

**DOI:** 10.1371/journal.pone.0149390

**Published:** 2016-02-16

**Authors:** Sarah Forbes, Joe Latimer, Prem K. Sreenivasan, Andrew J. McBain

**Affiliations:** 1 Manchester Pharmacy School, The University of Manchester, Manchester, United Kingdom; 2 Colgate-Palmolive Technology Center, Piscataway, NJ, United States of America; Medical University of South Carolina, UNITED STATES

## Abstract

Dentifrices can augment oral hygiene by inactivating bacteria and at sub-lethal concentrations may affect bacterial metabolism, potentially inhibiting acidogenesis, the main cause of caries. Reported herein is the development of a rapid method to simultaneously measure group-specific bactericidal and acidogenesis-mitigation effects of dentifrices on oral bacteria. Saliva was incubated aerobically and anaerobically in Tryptone Soya Broth, Wilkins-Chalgren Broth with mucin, or artificial saliva and was exposed to dentifrices containing triclosan/copolymer (TD); sodium fluoride (FD); stannous fluoride and zinc lactate (SFD1); or stannous fluoride, zinc lactate and stannous chloride (SFD2). Minimum inhibitory concentrations (MIC) were determined turbidometrically whilst group-specific minimum bactericidal concentrations (MBC) were assessed using growth media and conditions selective for total aerobes, total anaerobes, streptococci and Gram-negative anaerobes. Minimum acid neutralization concentration (MNC) was defined as the lowest concentration of dentifrice at which acidification was inhibited. Differences between MIC and MNC were calculated and normalized with respect to MIC to derive the combined inhibitory and neutralizing capacity (CINC), a cumulative measure of acidogenesis-mitigation and growth inhibition. The overall rank order for growth inhibition potency (MIC) under aerobic and anaerobic conditions was: TD> SFD2> SFD1> FD. Acidogenesis-mitigation (MNC) was ordered; TD> FD> SFD2> SFD1. CINC was ordered TD> FD> SFD2> SFD1 aerobically and TD> FD> SFD1> SFD2 anaerobically. With respect to group-specific bactericidal activity, TD generally exhibited the greatest potency, particularly against total aerobes, total anaerobes and streptococci. This approach enables the rapid simultaneous evaluation of acidity mitigation, growth inhibition and specific antimicrobial activity by dentifrices.

## Introduction

Dental plaque is a taxonomically diverse microbial community, which plays an important role in oral health and disease [1–3]. The establishment of oral diseases such as dental caries has been associated with changes in the taxonomic composition and metabolism of the oral microbiota [3], often in response to exogenous factors such as an excess of dietary fermentable sugars or poor dental hygiene resulting in inadequate plaque control [4,5]. The fermentation of dietary sugars by oral bacteria produces acids that reduce plaque pH and, if sustained, can demineralize tooth enamel forming lesions on the tooth surface, resulting in dental caries [6–8]. Evidence suggests that the development of caries is linked to shifts in bacterial community composition and increased acidogenesis [9–11]. Molecular characterization of the oral microbiota has revealed that there may be distinct bacterial communities associated with healthy compared to diseased oral cavities [1, 12], with caries being associated with increases in the abundance of acidogenic microorganisms including *Streptococcus mutan*s and homofermentative lactobacilli [3, 6].

The control of dental plaque by the routine brushing with dentifrice can improve oral hygiene, reducing the incidence of oral disease [13,14]. Antimicrobials commonly-used as adjuncts to oral hygiene such as triclosan [15], zinc citrate and stannous fluoride [16] have shown marked activity against oral bacteria. Triclosan (2,4,4’-trichloro-2’-hydroxydiphenyl ether), a chlorinated bisphenol, can inhibit bacterial synthesis of fatty acids at bacteriostatic concentrations by interacting with an NADH-dependent enoyl-acyl carrier protein reductase, FabI [17]. At higher concentrations bactericidal activity has been associated with direct effects on the bacterial cell membrane [18]. In oral pathogens such as *Streptococcus mutans*, which lack FabI, triclosan may also inhibit glycolysis, leading to a reduction in the production of harmful acids [19]. Zinc salts and stannous fluoride reportedly inhibit bacterial acid production by impairing glycolysis and carbohydrate fermentation [20,21]. These compounds have growth-inhibitory properties largely due to inhibitory effects on enzymes required for bacterial metabolism and growth [20, 21]. The pH of dental plaque is influenced by a variety of factors, most notably by bacterial acid production, which may be offset to some extent by the buffering effects of saliva [10]. The mitigation of acid production in saliva and plaque as well as the control of bacterial growth by dentifrices may therefore be advantageous in the maintenance of oral health.

Several variables are of interest in the pre-clinical assessment of oral care formulations. From a microbiological perspective these include the extent of bacteriostatic and/or bactericidal activity, which can be tested with pure or mixed cultures of oral bacteria. Assessing differential antimicrobial effects on the oral microbiota normally necessitates growing mixed oral bacteria in various *in vitro* systems, combined with differential viable counts. Investigating the functional effects of dentifrices (for example, the inhibition of acidogenesis or proteolysis) at sub-lethal concentrations is normally achieved (for acidogenesis inhibition) by exposing oral bacteria to diluted formulations and assessing changes in pH with a microelectrode, or by the chemical analyses of fermentation acids. Whilst such approaches have proven utility, a rapid and reproducible endpoint-based method for high-throughput testing of these variables would be a useful addition to the currently available methodology. Here we report the development of a method for assessing both acidogenesis-mitigating and antimicrobial effects of dentifrices against salivary microorganisms.

## Materials and Methods

### Chemical reagents and bacterial growth media

Chemicals were obtained from Sigma (Dorset, UK) and bacteriological media from Oxoid (Basingstoke, UK), unless otherwise stated. Bacterial growth media were sterilized at 121°C, 15 psi for 15 min prior to use. The dentifrice formulations evaluated were Colgate^®^ Total^®^ (TD), containing the actives triclosan (0.3% w/v), copolymer (2% w/v) and sodium monofluorophosphate (0.76% w/v); Colgate^®^ Cavity Protection^®^ (FD), containing, sodium monofluorophosphate (0.76% w/v); Crest^®^ Pro-Health^®^ (SFD1), containing the actives stannous fluoride (0.454% w/v) and zinc lactate (2.5% w/v) and Crest^®^ Pro-Health Clinical^®^ (SFD2), containing the actives stannous fluoride (0.454% w/v), zinc lactate stannous chloride (2.5% w/v). Dentifrices were suspended in sterile broth to produce slurries (50 mg ml^-1^). Test media included; artificial saliva (AS), Wilkins Chalgren with 2% mucin (WCM) and Tryptone Soya broth (TSB). Artificial saliva growth medium (AS) comprised (g/L) in distilled water: Mucin (type II, porcine, gastric), 2.5; bacteriological peptone, 2.0; tryptone, 2.0; yeast extract, 1.0; NaCl, 0.35; KCl, 0.2; CaCl_2_, 0.2; cysteine hydrochloride, 0.1; haemin, 0.001; vitamin K1, 0.0002 [22].

### Minimum inhibitory concentrations (MIC)

MICs were determined using the microdilution method as described previously [23]. Briefly, individual samples of fresh human saliva from 3 healthy volunteers were immediately diluted 1 to 100 in sterile broth (AS, WCM or TSB). Inoculated media (200μL) containing graded concentrations of the relevant dentifrice were incubated in the wells of a 96 well microtiter plate at 37°C (24 h) with agitation (100 rpm) in aerobic or anaerobic conditions. Anaerobic conditions (gas mix: 90% N_2_, 10% CO_2_ and 10% H_2_) were generated using an MG1000 anaerobic workstation (Don Whitley Scientific, Shipley, UK). The MIC was defined as the lowest concentration at which bacterial growth was inhibited. Growth was measured as light absorbance (496 nm) in comparison to an uninoculated well containing the equivalent concentration of dentifrice (negative control) and was detected using a microtiter plate reader (Powerwave XS, BioTek Instruments, Potton, UK).

### Minimum bactericidal concentrations (MBC)

A transferable solid phase screening system (Nunc, Denmark) was used to aseptically transfer media (approximately 3–4 μL) from the MIC plate wells to a 96 well-plate containing 200 μL of one of the following bacteriological media; Wilkins Chalgren Agar (with and without anaerobic Gram-negative supplement) or yeast extract, cysteine, sucrose agar [24] (TYCS; total streptococci). Plates were incubated at 37°C either aerobically or anaerobically (gas mix: 90% N_2_, 10% CO_2_ and 10% H_2_; MG1000 anaerobic workstation; Don Whitley Scientific, Shipley, UK). The MBC was defined as the lowest concentration of dentifrice at which no visible growth occurred after 3 d of incubation.

### Minimum acid neutralizing concentrations (MNC)

Phenol red pH indicator solution (10 μl) was added directly to each MIC plate well after incubation to determine acidification of the growth media. Plates were incubated for a further 15 min at room temperature before determination of colour change. The MNC was defined as the lowest concentration at which no colour change (red to yellow) occurred. The pH of each dentifrice was determined at all test concentrations using a pH meter with a needle electrode (Jenway, Staffordshire, UK).

### Combined inhibitory neutralizing capacity (CINC)

The CINC was calculated as follows; CINC = (MIC-MNC)/MIC. The CINC therefore provides a numerical value that represents the cumulative ability of a particular dentifrice to prevent bacterial acidogenesis below growth inhibitory concentrations in addition to its ability to completely inhibit bacterial growth. A higher CINC value would therefore indicate better overall acid mitigation activity and growth inhibitory activity when compared to a product with a lower CINC.

### Statistical analysis

Data (MIC, MBC and MNC) were analyzed using a one-way ANOVA (analysis of variance) and post-hoc Tukey analysis. MICs were directly compared to MNCs for individual formulations using a paired student’s t-test. In both cases P<0.05 was deemed to be statistically significant.

### Ethics statement

Advice was taken from the Chair of a University of Manchester Research Ethics Committee regarding the correct procedures associated with the use of human saliva samples. The committee granted exemption from formal ethics approval due to the nature of the work but as advised, informed written consent was obtained from all volunteers and all samples were collected anonymously.

## Results

### Specificity of dentifrices for different functional groups of oral bacteria

**Fig 1:**
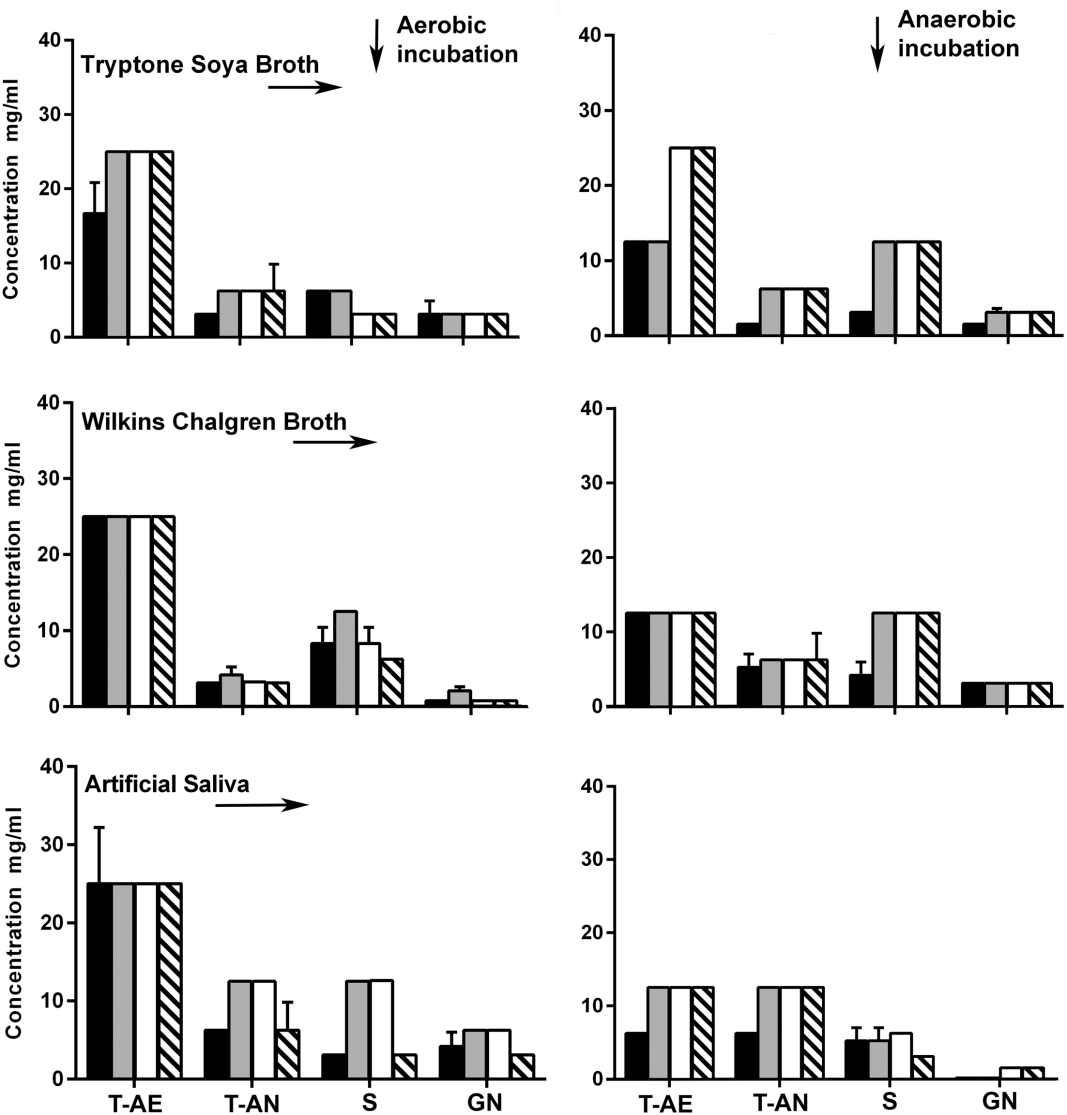
Minimum group-specific minimum bactericidal concentrations of dentifrices TD (black), FD (grey), SFD1 (white) and SFD2 (hatched) against salivary microorganisms tested in Tryptone Soya Broth, Wilkins Chalgren Broth with mucin or Artificial Saliva under aerobic or anaerobic conditions. Data show group-specific bactericidal activity for total aerobes (T-AE), total anaerobes (T-AN), Streptococci (S) and Gram-negative anaerobes (GN). Error bars represent standard errors for three biological replicates, each with three technical repeats. Statistical significance was determined by ANOVA with post-hoc Tukey analysis (P<0.05).

The bactericidal efficacy of the dentifrice formulations was assessed against total aerobes, total anaerobes, streptococci and Gram-negative anaerobes. Overall, TD showed significantly greater bactericidal activity (MBC; 6.3–25 mg/ml) when compared to the other test dentifrices against total aerobes P<0.05 (Fig 1), while FD, SFD1 and SFD2 demonstrated significantly higher MBCs of 12.5–25 mg/ml in all test media (and therefore lower potency). TD also exhibited an overall significantly higher bactericidal activity against total anaerobic bacteria (P<0.05) (Fig 1) with MBC values ranging between 1.6 and 6.3 mg/ml. Comparatively the potencies of SFD1 (MBC; 3.3–12.5 mg/ml), SFD2 (MBC; 3.3–12.5 mg/ml) and FD (MBC; 4.2–12.5 mg/ml) were not significantly different against anaerobic bacteria.

When incubated aerobically, MBCs for SFD2 against streptococci ranged between 3.1 and 6.3 mg/ml whilst values for TD ranged between 3.1 and 8.3 mg/ml, both were therefore potent but there was no statistically significant difference between MBCs for these two formulations (P>0.05). Equal or lower bactericidal activity against streptococci was observed for SFD1 (MBC; 3.1–12.5 mg/ml) and FD (MBC; 6.3–12.5 mg/ml). Under anaerobic conditions bactericidal activity against streptococci of TD (MBC; 3.1–5.2 mg/ml) was also significantly higher (P<0.05) than that of SFD2 (MBC; 3.1–12.5 mg/ml), FD (MBC; 5.2–12.5 mg/ml) and SFD1 (MBC; 6.3–12.5 mg/ml; Fig 1).

Bactericidal activity against Gram-negative anaerobes varied depending on incubation conditions. However, there was no significant difference in activity between all test treatments with MBC values ranging under aerobic incubation: SFD2 (0.8–3.1 mg/ml), TD (0.8–4.2 mg/ml), SFD1 (0.8–6.3 mg/ml) and FD (2.1–6.3 mg/ml; Fig 1) and under anaerobic incubation; TD (MBC; 0.2–3.1 mg/ml) FD (0.2–3.1 mg/ml), SFD1 (1.6–3.1 mg/ml) and SFD2 (1.6–3.1 mg/ml; Fig 1). Overall, MBCs were lower when tested in TSB when compared to values obtained in WCM or AS under aerobic conditions whilst MBC were lowest in AS under anaerobic conditions.

### Growth inhibition and acidogenesis-mitigating effects of dentifrices

**Fig 2:**
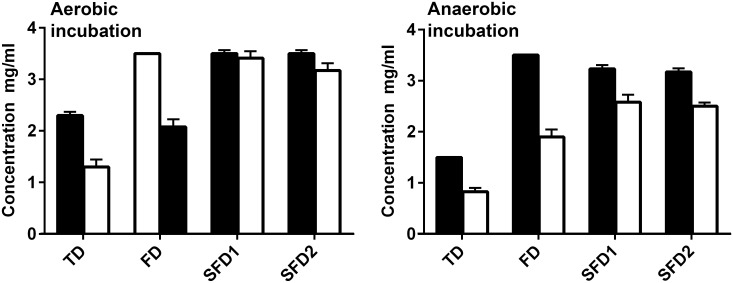
Minimum inhibitory concentrations (black bars) and minimum neutralizing concentrations (white bars) of test dentifrices (TD, FD, SFD1 and SFD2) in Tryptone Soya Broth under aerobic and anaerobic conditions. Error bars represent standard errors for three biological replicates, each with three technical repeats. Asterisks indicate statistically significant differences (p< 0.05) between MIC and MBC determined using a paired Student’s t-test.

The inhibit of bacterial growth and acidogenesis by the test pastes was assessed simultaneously in TSB by determining MICs and MNCs. MNC was defined as the lowest concentration of a dentifrice that maintained a pH of >7.5 after 24 h, as determined with a pH indicator. The pH of test dentifrices was determined across the MNC range generating pH values for SFD1 (pH 7.21–7.24), SFD2 (pH 7.22–7.25), FD (pH 7.27–7.24) and TD (pH 7.27–7.25). There was no significant difference in pH between the formulations at any test concentration (P<0.05). MNCs were significantly lower than MICs for both TD and FD (P<0.05) when incubated aerobically and for all dentifrices when incubated anaerobically (Fig 2). The rank order of MNC activity (mg/ml) was TD > FD > SFD2 > SFD1 under both aerobic and anaerobic conditions (Fig 2A), with no significant difference between SFD2 and SFD1 when incubated aerobically and FD, SFD2 and SFD1 when incubated anaerobically (P<0.05).

**Fig 3:**
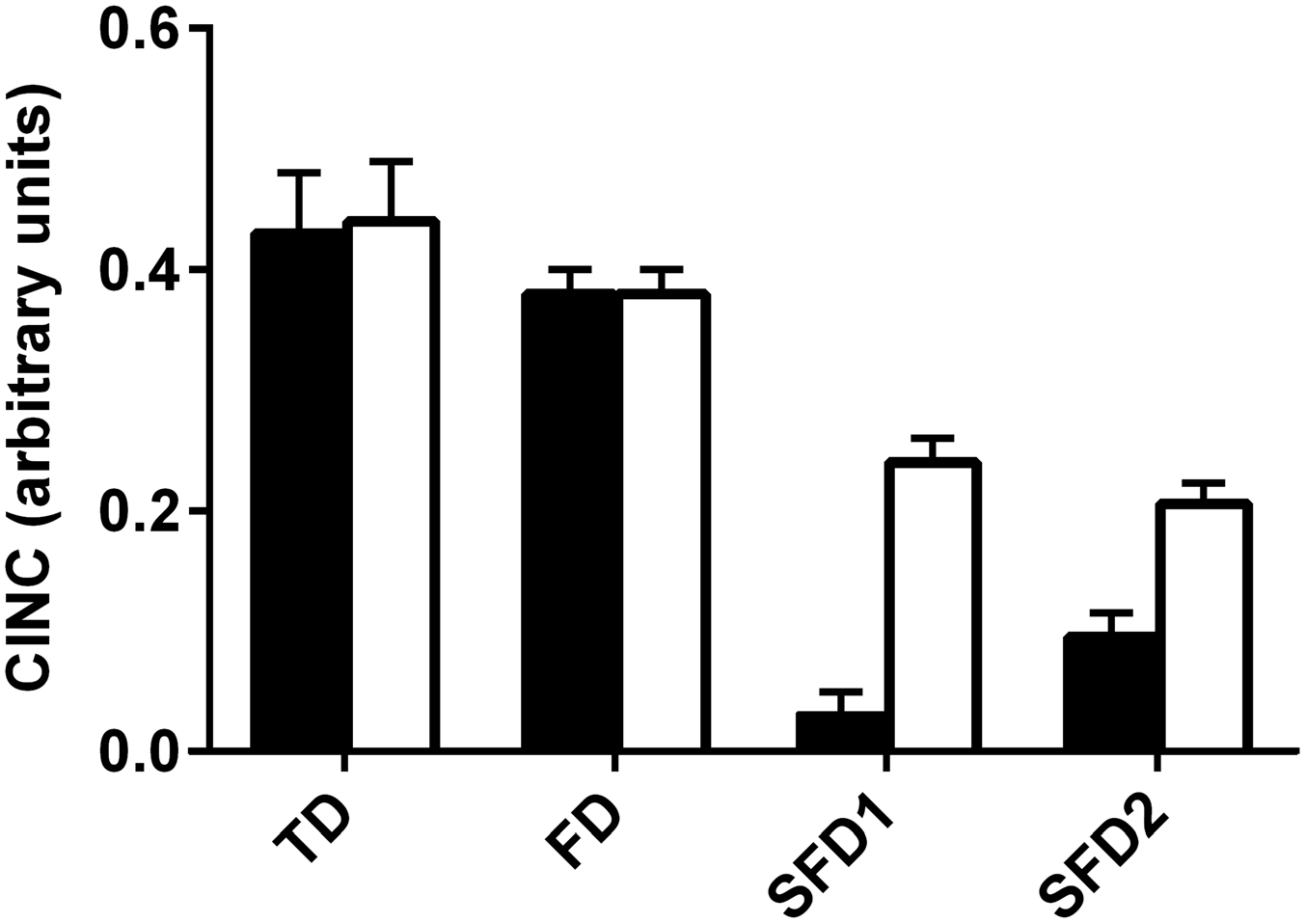
The combined inhibitory and neutralizing capacity (CINC) of the test dentifrices (TD, FD, SFD1 and SFD2) in Tryptone Soya Broth under aerobic (black bars) and anaerobic conditions (white bars). Error bars represent standard error for three biological replicates, each with three technical replicates.

The difference between MIC and MNC for each test paste was calculated and normalized with respect to MIC. The resulting values reflect the combined inhibitory and neutralizing capacity (CINC) of the test dentifrices, with a high value indicating a good ability to inhibit both acid production and bacterial growth. The CINC is therefore a single numerical measure of both specific acidogenesis mitigation and the inhibitory activity of a particular dentifrice against oral bacteria, providing the means of a clear preclinical measure of the activity of each test formulation against acidogenic bacteria indicating potential anti-caries activity. The rank order for CINC was TD > FD > SFD2 > SFD1 under aerobic conditions and TD > FD > SFD1> SFD2 under anaerobic conditions (Fig 3). Whether incubated aerobically or anaerobically, there was no significant difference between TD and FD, or between SFD1 and SFD1. All other differences were significant, as calculated by ANOVA with Tukey’s post-hoc analysis (P<0.05).

## Discussion

The use of antimicrobial formulations to control the accumulation of oral bacteria and therefore to reduce plaque accumulation can markedly decrease the risk of the development of oral disease such as caries and gingivitis [14, 25, 26]. Direct bactericidal activity of dentifrices against oral bacteria is therefore clearly desirable, particularly if the antimicrobial compound in the dentifrice is effective against bacteria involved in the etiology of oral diseases. The anti-caries efficacy of a formulation may however be enhanced through specific effects on bacterial metabolism, including inhibition of the fermentation processes which lead to acid production [6–8]. In the current study a microtitre plate-based system was used to assess group-specific bactericidal effects and acidogenesis-mitigation by four dentifrices, TD (containing triclosan/copolymer), FD (containing sodium monofluorophosphate), SFD1 (containing stannous fluoride and zinc lactate) and SFD2 (containing stannous fluoride, zinc lactate and stannous chloride). The minimum neutralizing concentration (MNC) and the combined inhibitory and neutralizing capacity (CINC) are proposed as objective measures by which to quantify specific effects of dentifrice formulations on bacterial metabolism in relation to acidogenesis-mitigation.

TD exhibited greatest bactericidal activity against total aerobes and total anaerobes during MIC testing, prior to plating on selective agars. These findings are in agreement with previous studies showing the broad-spectrum and high level of antibacterial activity of triclosan [27, 28]. TD also exhibited the highest overall level of bactericidal activity against streptococci when initially cultured anaerobically. Since the growth environment for plaque is predominantly anaerobic, the ability of a dentifrice to inactivate bacteria growing in anaerobic conditions may be advantageous to oral health. SFD2 demonstrated high overall bactericidal activity against streptococci when cultured aerobically. Previous investigations report the antibacterial activity of stannous fluoride against oral bacteria [29,30].

TD exhibited greater growth inhibitory effects against bacteria than FD, SFD1 and SFD2. MNC data also indicate that the test dentifrices inhibited acidogenesis at sub-inhibitory concentrations. This may be attributed to specific inhibitory effects on bacterial metabolism and subsequent acid production. For example, fluoride has been previously shown to inhibit sucrose-induced acid production in bacteria [30, 31], potentially accounting for some of the acidogenesis-mitigating activity observed in this study. However, despite the fact that TD and FD contain similar concentrations of fluoride, MNC data indicate that the acidogenesis-mitigating capability of TD was significantly greater than the other test dentifrices in both aerobic and anaerobic conditions. Furthermore, TD mitigated acidogenesis at concentrations considerably lower than those required for complete bacterial growth inhibition. Triclosan is known to function at bacteriostatic concentrations through inhibition of FabI, an enzyme involved in bacterial fatty acid synthesis [17], as well as through direct effects on the bacterial cytoplasmic membrane [18]. Importantly, triclosan may inhibit glycolysis and thus acid production in oral bacteria through inhibition of glycolytic enzymes such as pyruvate kinase, lactic dehydrogenase and aldolase, as well as via interference in the phosphoenolpyruvate: sugar phosphotransferase system [32, 33].

Both SFD1 and SFD2 demonstrated lower values for MNC than MIC, particularly when incubated anaerobically. In addition to stannous fluoride, which has been shown to reduce acid production in cariogenic bacteria [30, 31], both of these formulations contain zinc, a known inhibitor of glycolysis providing a plausible explanation for the acidogenesis-mitigating capabilities of these formulations at sub-growth inhibitory concentrations for oral anaerobic bacteria. CINC values facilitated the comparison of the dentifrices on the basis of both their growth inhibitory effects and the inhibition of acidogenesis at sub-lethal concentrations. When combining the effects of growth-inhibition and acidogenesis-mitigation, TD demonstrated the highest combined inhibitory and neutralizing capacity (CINC). This is probably due to the bactericidal activity of triclosan, combined with inhibitory effects on bacterial glycolysis [27, 32]. This is a potentially beneficial characteristic of a dentifrice and may partly explain the clinical efficacy of triclosan-containing dentifrices in reducing plaque. Under aerobic conditions SFD2 demonstrated higher CINC activity than SFD1, due to its lower MNC value. This suggests that the addition of stannous chloride into dental formulations could increase the acidogenesis inhibitory activity in oral bacteria.

The capacity of a dentifrice to inhibit plaque acidogenesis is an important property of oral healthcare formulations. Objective criteria to express the minimum concentration of dentifrice required to mitigate acidogenesis in controlled conditions (MNC), and the combined inhibitory and neutralizing capacity (CINC) may therefore be used in the pre-clinical evaluation of dentifrices, enabling acidity mitigation, growth inhibition and specific antimicrobial activity to be determined simultaneously in a robust and reproducible fashion.

## Conclusion

The etiology of dental caries primarily involves acid production by oral bacteria and therefore the reduction of bacterial acidogensis; whether mediated through the inactivation of bacteria, the inhibition of bacterial growth or by specific effects on bacterial metabolism, is a desirable activity for oral health products. In the current investigation a method through which acidity mitigation, growth inhibition and specific antimicrobial activity can be determined simultaneously is described. Test formulations used to evaluate the method exhibited marked differences in their growth-inhibitory and group-specific bactericidal activities. Acidogenesis-mitigation was observed at concentrations lower than required for bacterial growth inhibition. A dentifrice containing triclosan (TD) exhibited a higher level of antimicrobial activity and acidogenesis-mitigating ability than other test dentifrices.
